# Fruit and Vegetable Intake in Adolescents: Association with Socioeconomic Status and Exposure to Supermarkets and Fast Food Outlets

**DOI:** 10.1155/2012/185484

**Published:** 2012-09-04

**Authors:** Chalida M. Svastisalee, Bjørn E. Holstein, Pernille Due

**Affiliations:** ^1^National Institute of Public Health, University of Southern Denmark, Øster Farimagsgade, 5A, 1353 Copenhagen K, Denmark; ^2^Department of Social Medicine, University of Copenhagen, Øster Farimagsgade, 5, P.O. Box 2099, 1014 Copenhagen K, Denmark

## Abstract

*Background*. We investigated differences in family social class associations between food outlet exposure and fruit and vegetable intake. *Methods*. We supplemented data from the 2006 Health Behavior in School Aged Children Study (*n* = 6, 096) with geocoded food outlet information surrounding schools (*n* = 80). We used multilevel logistic regression to examine associations between infrequent fruit and vegetable intake and supermarket and fast food outlet concentration, stratified by family social class. *Results*. Boys and older children were most likely to eat fruit and vegetables infrequently. High fast food outlet exposure was marginally significant for low fruit intake in low social class children only. Children from middle and low social class backgrounds attending schools with combined high fast food outlet/low supermarket exposure were most likely to report infrequent fruit intake (OR_low_ = 1.60; CI:  1.02–2.45; OR_mid_ = 1.40; CI:  1.03–190). Children from low social class backgrounds were also likely to report infrequent vegetable intake, given low supermarket and high fast food outlet exposure (OR = 1.79; CI:  0.99–3.21). *Conclusion*. Our findings suggest social class modifies the relationship between intake and food outlet concentration. School interventions improving fruit and vegetable intake should consider neighborhood surroundings, targetting older children from low social class backgrounds.

## 1. Introduction 

Fruit and vegetable intake is an important indicator of diet quality and protective against certain chronic diseases [1]. Despite these benefits, adolescents consume a disproportionate amount of nutrient poor foods, such as fast food or snack foods [2, 3] and do not meet dietary guidelines for fruit and vegetables [4–8]. 

Individual sociodemographic correlates of low fruit and vegetable intake are well known [9]. Boys tend to consume less fruit and vegetables than girls, while intake levels decline with increasing age and decreasing socioeconomic status [9]. 

Concerns about low fruit and vegetable intake, especially among those from low income backgrounds, have prompted scholars to examine whether a lack of access to food outlets selling fruit or vegetables may be a problem. Various studies in North America [10–15] and one study in New Zealand [16] demonstrate schools are generally within walking distance to places where calorie dense, nutrient poor foods are usually sold, such as fast food restaurants or convenience stores. These food outlets are often placed in lower income neighborhoods [12, 13, 15, 17]. Such findings prompt strong argumentation for extending promotional strategies beyond school borders by considering the food environment in surrounding school neighborhoods as part of the broader framework for the creation of healthy eating zones [18, 19]. 

 Although proximity of fast food outlets placed near schools may influence consumption of unhealthy foods, there is still little evidence available. There are few published studies that examine the relationship between dietary behavior in youth and exposure to food outlets in the school neighborhood. A US study [14] reported decreased fruit and vegetable consumption with increased fast food outlet exposure, while another study in the US found no relationship between school neighborhood environments and diet in adolescents [20]. In the UK, supermarket density was associated with increased vegetable intake but also unhealthy foods [21]. Lastly, a study in The Netherlands found little relationship between food outlets and soft drink or snack consumption [3]. In summary, the available studies do not show a coherent association between food intake and exposure to food outlets. 

 These studies, in particular, included objective geographic measures to ensure representation of the food environment. However, individual socioeconomic characteristics, while controlled in analyses [14, 21], were not fully explored in combination with the food environment. Socioeconomic position may have an influence on where one may be able to afford to live. Further, socioeconomic position may moderate the association between exposure to food outlets and students' intake of fruit and vegetables [22]. For instance, lower intake of fruit and vegetables may result from low social class backgrounds given high exposure to fast food outlets, while high social class may serve as a buffer against effects of high fast food outlet exposure. To our knowledge, there are no published studies of the association between exposure to food outlets and dietary behavior in a Scandinavian context. In particular, the unique socioeconomic structure lends itself to less income disparity, as income tends to be redistributed through taxation schemes [23]. This is also evident in Denmark's Gini coefficient of 0.25, indicating smaller differences between wealthy and nonwealthy residents [24]. In comparison, the US has a Gini coefficient of 0.41, indicating larger differences between those with low and high incomes [25]. 

Therefore, the first objective of this paper was to examine the association between infrequent fruit and vegetable intake in Danish youth and exposure to food outlets in school neighborhoods. We analysed exposure to supermarkets and fast food outlets separately and in combination. The second objective was to examine whether individual social class background modified the association between supermarket and fast food outlet concentration and intake; that is, we analysed the above associations in models stratified by family social class. 

## 2. Methods

### 2.1. Design and Study Population

Data were drawn from the Danish contribution to the international Health Behavior in school-aged children study (HBSC), which is a cross-sectional examination of health and health behaviors in 11-, 13-, and 15-yr-old children. Students participate in a nationally representative sample of schools [26]. 

In 2006, 100 randomly selected schools were invited to partake in the Danish HBSC Study. Eighty of these schools consented to participation while 20 declined participation because of participation in other studies. Of 7,043 students enrolled in grades 5th, 7th, and 9th (corresponding ages: 11, 13, and 15 years of age), a total of 6,269 (89% response rate) participated in the study. Students answered the internationally standardized HBSC questionnaire [27] during one class period. 

In Denmark, there is no formal body for ethical assessment of questionnaire surveys, but we ensured that study protocol complied with the Helsinki II declaration. Study participation was voluntary, with prior permission granted by the school leaders, the parents' school boards, and the student boards. All surveys were conducted anonymously, identifiable only by participant number, making it unfeasible to compare differences between participants and nonparticipants.

## 3. Measures

### 3.1. Exposure Variables

In Denmark, local schools house all levels from kindergarten to the 10th grade, which typically spans children between the ages of 6 to 16 years. Over 60% of Danish adolescents (13–15 yr) cycle to and from school for a duration of 20 minutes per day [28], suggesting that children attend school where they live, and information about the school neighborhood may reflect area of residence. For the purpose of this analysis, we obtained from the Danish Tax Registry a total of 2,358 addresses for fast food outlets and supermarkets, bounded by school postal codes (ave area: 59 km^2^ (SD: 70 km^2^)) to initially ensure that all outlets surrounded study schools. Although we use the postal code of each school as the initial sampling area of food outlets, our main area of study employs the use of radial buffers around the schools to illustrate food outlets in the school area are highly accessible [3, 10, 11]. All food outlets were established in 2006 or earlier and categorized by European standardized business codes (Nomenclature des Activites Economiques codes, NACE) [29]. We geocoded all addresses using ArcGIS (ESRI 9.2) and categorized them into two distinct categories: (1) retail supermarkets (includes all retail, discount, large chain, and small independent supermarkets and grocers; *n* = 1,078) and (2) fast food outlets and grill bars, which were also identified by their own unique NACE code (*n* = 1,280). In order to examine validity of address listings, we used ground-truthing methods [30] to check for the absence and presence of addresses and determined levels of concordance using percent overlap, positive predictive value (PPV), and sensitivity between information provided by each source and field validation: Teledanmark (*n* = 2,358), Stockmann (*n* = 863), and Krak.dk (*n* = 1,424). Overall, we report moderate percent overlap between the Tax Registry (65%), Teledanmark (70%), but high overlap using Stockmann (90%) and Krak.dk (82%). PPV was good to excellent [31] (range: 0.80–0.96), while sensitivity was moderate to excellent (range: 0.72–0.92). 

Using ArcGIS, we extracted total number of supermarkets (*n* = 37) and fast food outlets (*n* = 84), as well as road segments (kilometers) contained within 300-meter radial buffers from each school. Previous distance measures used in ecological studies examined ranges between 400 and 800 meters from the school [3, 10, 11, 14, 21]. We chose 300-meters to reflect half of the Danish planning standard for foot transport from any public transportation stop to a public facility [32], as well as simulate a 5-minute walk for children [21] accessible from school. Furthermore, we found no substantial differences in association with intake when we considered 600 meters from the school (data not shown). 

Exposure was calculated by food outlet concentration as the number of food outlets divided by total road segments [33] within 300 meters from the school. We tested the association between crude number of supermarket and fast food outlets and the pupils' intake of fruit and vegetables (data not shown). The pattern of associations from this crude analysis did not differ from findings presented in the paper. The use of retail concentration by road availability may be much more effective in characterizing urban environments, because it accounts for distribution of retail places within an allotment of space [34] and potentially illustrates how such places are framed within a certain social context [35]. The use of retail concentration measures by road availability has proven successful in demonstrating the association between neighbourhood concentration of package stores with violent assaults [36] and with motor vehicle accidents [35] but has not been used to characterize retail outlet exposure around schools and dietary intake. Thresholds for exposure were based on visual plots of the number of food outlets by concentration, as well as by distributions. For supermarkets, the average exposure was 0.142 outlets/km (SD = 0.26). For fast food outlets, the average exposure was 0.273 outlets/km (SD = 0.41). The low absolute number of supermarkets and fast food outlets (see Table 1), observations from the visual plots, and the nonnormal distribution of the exposure variables suggested that it was difficult to treat the exposure variables as linear ones. Therefore, we decided to dichotomise the variables. High fast food outlet exposure was defined as two or more fast food outlets per total road kilometers, while high supermarket exposure was defined as more than one supermarket per total road kilometers. We formulated a variable which combined exposure to supermarkets and fast food outlets within four categories: high-low, low-high, low-low, and high-high. 

### 3.2. Outcome Measures

We used student responses to two food frequency questions for two outcome measures regarding fruit and vegetable intake. Students were separately asked how frequently they consumed (a) fruit and (b) vegetables (response key: 1 = never, 2 = less than 1 time/week, 3 = 1 time/week, 4 = 2–4 d/week, 5 = 5-6 d/week, 6 = once/d, 7 = more than once daily). Category responses for the HBSC questionnaire have been validated by food frequency measures designed to monitor habitual intake [37]. We defined infrequent intake of fruit and vegetables as less than daily intake (categorical responses 1–5), as we wanted to examine the risk of not meeting Danish nutritional guidelines of daily intake of fruit and vegetables [38]. A total of 96 students were excluded from analysis due to missing information on either of the two outcome measures.

### 3.3. Covariates

We tested individual variables (age, sex, and family social class) for associations with fruit and vegetable intake [9]. Family social class was determined by student report of job title and place of work of the mother and father. Each occupation was coded into one of five social class variables (I = high to V = low) using standards similar to the UK Registrar General classification [39]. Each student was classified by highest ranking parental occupation into family social classes I to V. We added family social class VI for children providing occupation information that was unclassifiable but knew the parent as active in the workforce. Family social class VII was constructed for students from families whose parents were absent from the workforce and living from social welfare benefits. 

We recoded social class into high (I-II), middle (III-IV), and low (V and VII) and excluded students if they were completely missing family social class information (*n* = 139). 

In the analyses employing the first research question, we kept the unclassified social class (VI) in the analyses as a separate category in order to ensure the statistical power of the study. In the analyses of the second research question (stratified analyses) we included only children from high, middle, and low social class backgrounds and excluded social class category VI for which social class was uncertain. 

## 4. Statistical Analyses

The analysis was based on 80 schools and 6,034 students with complete data on all covariates and outcome measures. Statistical analyses were conducted in SAS 9.2 (SAS Institute, Cary, NC, USA). We determined baseline intake prevalences and tested for differences between boys and girls, between grades, and between family social classes using *χ* ^2^ statistics. Spearman correlation analyses were performed to ensure supermarket and food outlet exposure variables were independently distinct from each other (*ρ*: 0.36). 

To analyze infrequent fruit and vegetable intake in students, as posed in the first objective, we used logistic multilevel regression analysis (Proc Glimmix), allowing for random school effects to account for clustering of students within schools. There were no substantial differences in odds ratios for infrequent fruit and vegetable intake in sex-stratified analyses (data not shown). As a result, we report results for boys and girls together.

In order to answer the second research question, we conducted the analyses stratified by family social class to differentiate association patterns across socioeconomic strata. Preliminary analyses also showed different association patterns across socioeconomic strata (data not shown). In stratifying the analysis, we separate potential effects of confounding by social class, but we are still able to examine individual and combined effects of the exposures [40]. Additionally, stratification allows us to view potentially protective effects of higher social class on reducing likelihood of low fruit and vegetable intake. We tested each of the two exposure variables (exposure to supermarkets, exposure to fast food outlets) separately to determine the association with each of the two outcome variables: individual fruit intake and individual vegetable intake, mutually controlling for sex and grade. Finally, we tested the association between infrequent fruit and vegetable intake and the variable which combine supermarket and fast food outlet concentration. These analyses were also adjusted for sex and grade. 

## 5. Results

In Table 1, high prevalences of infrequent intake of fruit and vegetables were more common among boys than girls, more common among 7th and 9th graders than among 5th graders and more common among students from low/middle social classes than high social classes. Age- and grade-adjusted logistic regression analysis (data not shown) also confirmed the association between family social class and infrequent fruit intake. The estimates for middle family social class versus high was 1.66 (CI: 1.45–1.89), low versus high 1.46 (CI: 1.24–1.71), and unclassifiable social class versus high 1.68 (CI: 1.43–1.99). Corresponding OR values for infrequent vegetable intake by family social class were 1.59 (CI: 1.39–1.81), 1.86 (CI: 1.58–2.19), and 1.73 (CI: 1.47–2.05).

Results from *χ* ^2^ tests in Table 1 also show that students attending schools in areas with low supermarket exposure were significantly more likely to have an infrequent intake of fruit and vegetables. The exposure to fast food outlets was also associated with students' intake of fruit and vegetables. 

Tables 2 and 3 address the second research question, whether individual social class background modifies the association between intake and exposure to supermarkets and fast food outlets. Both tables present fully adjusted results from multilevel logistic regression analyses.

Table 2 shows an association between low supermarket exposure and infrequent fruit intake but only marginally significant in the lower social classes (OR = 1.32; CI: 0.98–1.76). The combination of low supermarket exposure and high fast food outlet exposure, however, was associated with infrequent fruit intake among children from lower social class backgrounds (OR = 1.60; CI: 1.02–2.45). We observed significantly higher ORs for infrequent fruit intake among boys of all social classes (OR_low_ = 1.80; CI: 1.42–2.30; OR_mid_ = 1.82; CI: 1.54–2.14; OR_high_ = 1.60; 1.21–1.98). Older students in grades 7 and 9 from low social class backgrounds (OR_7th_ = 1.32; CI: 1.00–1.74; OR_9th_ = 1.88; CI: 1.38–2.57) and 9th grade students in the high social class (OR = 1.50; CI: 1.13–1.99) were more likely to report infrequent fruit intake. 

Table 3 shows marginal significance for infrequent vegetable intake with combined low supermarket and high fast food outlet exposure in low-income children (OR = 1.79; CI: 0.99–3.21). There was no association between exposure to fast food outlets or supermarkets and infrequent vegetable intake. Within all family social classes, boys had significantly increased OR for infrequent vegetable intake than girls (OR_low_ = 1.37; CI: 1.06–1.78; OR_mid_ = 1.49; CI: 1.26–1.76; OR_high_ = 1.53; CI: 1.23–1.90). In the lower social classes but not in the middle and high social classes, students in the 7th and 9th grade had higher OR for infrequent vegetable intake than students in the 5th grade (OR_7th_ = 1.51; CI: 1.12–2.03; OR_9th_ = 1.59; CI: 1.14–2.21).

## 6. Discussion

We report two main findings. First, older children, especially 9th grade students within low social class backgrounds, have highest odds for infrequent fruit and vegetable intake. Second, children within low social class backgrounds have greatest odds for infrequent fruit and vegetable consumption in environments with combined low exposure to supermarkets and high exposure to fast food outlets. Exposure to fast food outlets was not in itself associated with infrequent fruit and vegetable intake, neither in the full sample nor in any of the social class strata. A potential interpretation of these findings is that middle and high social class background acts as a buffer against the effect of exposure to an imbalance of food outlets in the neighborhood. 

 In general, our findings support other studies reporting low fruit and vegetable consumption in youth with fast food outlet exposure in school [14] or home environments [41, 42]. For example, Davis and Carpenter [14] found adolescents attending schools exposed to a fast food outlet within a half-mile radius consumed fewer servings of fruit and vegetables than those attending schools with no outlets in the vicinity. We found odds for infrequent fruit intake were greater with high fast food outlet exposure especially in low income children, suggesting a deleterious effect on fruit intake. 

It is not easy to compare the published studies because of differences in study population, definition of neighborhood size, and measurement of food environment characteristics. Study findings did not support reported associations with fruit and vegetable intake and food outlet density measures [21, 42]. Our findings do, however, corroborate with studies demonstrating a lack of relationship between distance to food outlets and dietary behavior in school neighborhoods [3]. 

Our results broadly illustrate that socioeconomic status should be considered when examining relationships between the exposure to food outlets and individual dietary intake. In our study, students from low social class were least likely to consume fruit when exposed to a combination of few supermarkets and many fast food outlets. 

Our approach was different from other studies examining the relationship between socioeconomic status, the built environment, and dietary intake, as our population sample was large enough to allow stratification by family social class. A similar comparison can be made in a US study of adults [43] reporting individual SES to have a differential effect on the built environment in relationship to BMI. The lack of association for fast food outlets or supermarkets alone with infrequent fruit and vegetable intake was surprising but may be attributed to distributional effects, as there were fewer students attending schools with high fast food exposure compared to students attending schools with high supermarket exposure [44]. The findings also illustrate the complexity that the food environment may play as barriers and enhancers of fruit and vegetable consumption. As we examined food outlet exposure 600 meters away from the school and found no relationship with fruit and vegetable intake, we do not believe that constructing a different measure further away from the school to accommodate more fast food outlets would change the association. 

### 6.1. Strengths and Limitations

Study merits include the use of a large representative sample of Danish youth as well as objective information to characterize aspects of exposure to food outlets in the school neighborhood. An additional merit of this study is that we were able to examine fruit and vegetable intake separately, as we acknowledge that each dietary component has unique correlates of intake [9].

Study limitations involve its cross-sectional design, of which we are unable to draw definitive conclusions about causality between the food environment and eating behavior. It is possible that availability of food outlets influences health-oriented families to choose their area of residence and therefore their school district. If this is the case, our associations are a result of selection rather than influence of food outlet exposure. 

Our analysis did not include all types of food outlets, such as green grocers, food kiosks, or full service restaurants. While we acknowledge that the presence of green grocers in a neighborhood could enhance fruit and vegetable consumption, there were very few outlets in our dataset that could be considered to impact intake. Nevertheless, this limitation may result in an underestimation of the relationship between food outlet exposure and fruit and vegetable intake. The study may also suffer from misclassification. Secondary sources of retail information may not be complete, although we made an effort to cross-reference our food retail database with other published sources of information. Despite good-to-high PPV measures, indicating a high degree of correspondence between data sources, we acknowledge misrepresentation may be unavoidable [45–47]. We also acknowledge the potential for reporting bias in using self-reported dietary outcome measures; however, the HBSC food frequency measure has been previously validated against seven-day food diary records with good correspondence between the two [37]. Additionally, we cannot ignore the potential for unmeasured confounding, that is, lack of proper explanatory variables. Area level SES, for instance, is another important confounder that may characterize the types of food outlets present within a neighborhood [15, 16, 48]. As the HBSC study is anonymous, and area level information at the school district level is linked to personal registry information in Denmark, we were unable to examine the contribution of area level SES to food outlet exposure. We also acknowledge that the eating environment within the school, for example, such as where students consume lunch, or fruit, and vegetable subscriptions [49], may also be related to intake level, and future research may include these factors as correlates of intake. Finally, our study is also based on certain assumptions that fast food intake and fruit and vegetable intake are inversely related [2, 50]. In this analysis, we were unable to control for fast food intake, which may be a potential confounder in the relationship between fast food retail concentration and fruit or vegetable intake [51, 52] and would consider fast food intake in future analyses. 

### 6.2. Implications

Our findings suggest that the combined effects of low supermarket with high fast food outlet exposure, however, are intriguing for future research. Future research should include further exploration of the entire food environment as a whole context and that there may be competing effects based on the spatial distribution of types of food outlets, of which certain groups may be more vulnerable to poor dietary behaviors.

Our analytical approach was very different from other studies examining the combined relationship of the food outlet exposure and socioeconomic status on adolescent fruit and vegetable intake. Our findings indicate a need to further explore the combined effects of contextual and socioeconomic factors. Future research interests should consider not only SES strata but perhaps even information on whether children visit the food outlets within the school neighborhood, during which time of the day, as well as purchasing and food consumption. Similar to studies in North America [10–15, 17], schools in Denmark also have access to a variety of food outlets, which could impact dietary behavior among children should they purchase foods during the free period or lunch break that could replace healthier food choices. As such, there is need to consider local areas around schools, especially if foods sold within close proximity to the school compete with school efforts to create an atmosphere conducive to health [19, 53]. 

## 7. Conclusion

Our findings show new results in that while family social class is an important predictor for infrequent fruit and vegetable intake in youth, the data also suggest that family social class modifies the relationship of food outlet exposure on intake. Children and older children from low family social class backgrounds and in areas of combined low supermarket and high fast food outlet concentration have the greatest odds of infrequent fruit intake. Implications of this study may contribute to the growing evidence base needed to establish or strengthen school policies for children and adolescents, particularly among families from low social class backgrounds.

## Figures and Tables

**Table 1:** Proportion of students with infrequent consumption of fruit or vegetables by sociodemographic characteristics and neighborhood variables.

	<Daily fruit intake		<Daily vegetable intake	
	(*n* = 3506/6034)		(*n* = 3791/6034)	
	%	*P*	%	*P*
All students, *n* = 6034	58.1		62.8	

Sex				
(i) boys (reference), *n* = 2950	65.0	<.0001	67.2	<.0001
(ii) girls, *n* = 3084	51.6		58.6	

Grade				
(i) fifth grade (reference), *n* = 2278	54.4	<.0001	60.2	0.0042
(ii) seventh grade, *n* = 2140	58.6		64.4	
(iii) ninth grade, *n* = 1616	62.8		64.5	

Family social class				
(i) high (reference), *n* = 1384	50.0	<.0001	53.9	<.0001
(ii) middle, *n* = 2478	61.3		64.3	
(iii) low, *n* = 1141	58.3		67.7	
(iv) unclassifiable, *n* = 1031	61.4		66.1	

Supermarket counts (300 meters)				
(i) 0, *n* = 55 schools, *n* = 3943 students	59.2	0.1569	63.7	0.1198
(ii) 1, *n* = 17 schools, *n* = 1379 students	55.7		61.1	
(iii) 2, *n* = 5 schools, *n* = 411 students	55.7		58.6	
(iv) 3, *n* = 2 schools, *n* = 255 students	58.0		66.3	
(v) 4, *n* = 1 school, *n* = 46 students	54.4		60.9	

Fast food outlet counts (300 meters)				
(i) 0, *n* =48 schools, *n* = 3388 students	57.0	0.0003	61.7	<.0001
(ii) 1, *n* = 17 schools, *n* = 1190 students	60.8		66.5	
(iii) 2, *n* = 5 schools, *n* = 530 students	61.1		66.2	
(iv) 3, *n* = 4 schools, *n* = 471 students	59.9		59.0	
(v) 4, *n* = 2 schools, *n* = 218 students	56.8		67.0	
(vi) 5 or more, *n* = 4 schools, *n* = 237 students	50.5 (ave)		55.4 (ave)	

Supermarket concentration (300 meters)				
(i) low, *n* = 55 schools, 3743 students	59.3	0.0176	63.8	0.0522
(ii) high, *n* = 26 schools, 2291 students	56.1		61.3	

Fast food outlet concentration (300 meters)				
(i) low, *n* = 56 schools, 4422 students	57.3	0.0519	62.0	0.0511
(ii) high, *n* = 24 schools, 1812 students	60.0		64.7	

**Table 2:** Adjusted odds ratios (95% CI) for infrequent *fruit* consumption, separate analyses for students in low, middle, and high family social classes.

	Low family social class, *n* = 1141OR (95% CI)	Middle family social class, *n* = 2478OR (95% CI)	High family social class, *n* = 1384OR (95% CI)
Sex^a^			
Boys versus girls	1.80 (1.42–2.30)***	1.82 (1.54–2.14)***	1.60 (1.21–1.98)***

Grade^a^			
7th versus 5th	1.32 (1.00–1.74)*	1.20 (0.99–1.46)	1.07 (0.83–1.38)
9th versus 5th	1.88 (1.38–2.57)***	1.16 (0.95–1.43)	1.50 (1.13–1.99)**

Supermarket exposure^a^			
low versus high	1.17 (0.89–1.54)	1.17 (0.97–1.40)	1.08 (0.80–1.45)

Fast food outlet exposure^a^			
high versus low	1.32 (0.98–1.76)	1.18 (0.97–1.43)	1.23 (0.89–1.69)

Interaction supermarket* fast food exposure^b^			
(i) high, low	1.0	1.0	1.0
(ii) low, high	1.60 (1.02–2.45)*	1.40 (1.03–1.90)*	1.45 (0.89–2.38)
(iii) low, low	1.10 (0.78–1.54)	1.11 (0.88–1.40)	0.91 (0.64–1.29)
(iv) high, high	1.17 (0.75–1.84)	1.09 (0.81–1.47)	0.94 (0.61–1.46)

^a^Odds ratios mutually adjusted for sex, grade, and supermarket and fast food outlet exposure.

^b^Adjusted for sex and grade.

Significant levels at *0.05, **0.01, ***0.001.

**Table 3:** Adjusted odds ratios (95% CI) for infrequent *vegetable* consumption, separate analyses for students in low, middle, and high family social classes.

	Low family social class, *n* = 1141OR (95% CI)	Middle family social class, *n* = 2478OR (95% CI)	High family social class, *n* = 1384OR (95% CI)
Sex^a^			
Boys versus girls	1.37 (1.06–1.78)*	1.49 (1.26–1.76)***	1.53 (1.23–1.90)***

Grade^a^			
7th versus 5th	1.51 (1.12–2.03)**	1.15 (0.94–1.40)	1.14 (0.88–1.45)
9th versus 5th	1.59 (1.14–2.21)**	1.01 (0.82–1.24)	1.18 (0.88–1.45)

Supermarket exposure^a^			
low versus high	1.33 (0.92–1.90)	1.19 (0.92–1.54)	1.04 (0.80–1.35)

Fast food outlet exposure^a^			
high versus low	1.17 (0.80–1.71)	1.20 (0.92–1.57)	1.26 (0.95–1.66)

Interaction supermarket * fast food exposure^b^			
(i) high, low	1.0	1.0	1.0
(ii) low, high	1.79 (0.99–3.21)	1.14 (0.76–1.73)	1.27 (0.84–1.92)
(iii) low, low	1.11 (0.71–1.74)	1.05 (0.71–1.74)	0.95 (0.69–1.30)
(iv) high, high	0.87 (0.50–1.53)	0.91 (0.58–1.42)	0.94 (0.62–1.43)

^a^Odds ratios mutually adjusted for sex, grade, and supermarket and fast food outlet exposure.

^b^Adjusted for sex and grade.

Significant levels at *0.05, **0.01, ***0.001.

## Abbreviations

HBSC: Health behaviors in school children study.
